# Vocal Feature Changes for Monitoring Parkinson’s Disease Progression—A Systematic Review

**DOI:** 10.3390/brainsci15030320

**Published:** 2025-03-19

**Authors:** Helen Wright, Vered Aharonson

**Affiliations:** 1 School of Electrical and Information Engineering, University of the Witwatersrand, Johannesburg 2050, South Africa; vered.aharonson@wits.ac.za; 2 Department of Basic and Clinical Sciences, Medical School University of Nicosia, CY-1700 Nicosia, Cyprus

**Keywords:** Parkinson’s disease, vocal features, progression, monitoring

## Abstract

**Background:** Parkinson’s disease has a significant impact on vocal characteristics and speech patterns, making them potential biomarkers for monitoring disease progression. To effectively utilise these biomarkers, it is essential to understand how they evolve over time as this degenerative disease progresses. **Objectives:** This review aims to identify the most used vocal features in Parkinson’s disease monitoring and to track the temporal changes observed in each feature. **Methods:** An online database search was conducted to identify studies on voice and speech changes associated with Parkinson’s disease progression. The analysis examined the features and their temporal changes to identify potential feature classes and trends. **Results:** Eighteen features were identified and categorised into three main aspects of speech: articulation, phonation and prosody. While twelve of these features exhibited measurable variations in Parkinsonian voices compared to those of healthy individuals, insights into long-term changes were limited. **Conclusions:** Vocal features can effectively discriminate Parkinsonian voices and may be used to monitor changes through disease progression. These changes remain underexplored and necessitate more evidence from long-term studies. The additional evidence could provide clinical insights into the disease and enhance the effectiveness of automated voice-based monitoring.

## 1. Introduction

Parkinson’s disease (PD) is a complex neurodegenerative disease characterised by the progressive loss of dopaminergic neurons in the substantia nigra pars compacta. The course of the disease may be influenced by many factors, including the development of Lewy bodies, genetic and environmental aspects and endocrine abnormalities [1]. These factors may also lead to the symptoms that are observed as the disease progresses.

A worsening of symptoms marks the progression of Parkinson’s disease over time. Clinical rating scales are used by healthcare professionals to capture the symptoms’ temporal patterns and to assess the disease progression. Historically, these scales focused on motor functions and physical disability but have been expanded to include non-motor aspects, such as cognitive functions [2,3,4,5,6]. This offers a more comprehensive understanding of the long-term effects of the disease and its underlying pathology [7]. Since the rating scales predominantly rely on observations by healthcare professionals, they are inherently subjective. The training, skills and experience of the examining person may bias the rating and potentially introduce inter- and intra-rater variability [8,9]. These assessments must be conducted in person, posing challenges for patients in remote locations or those in advanced stages of the disease. The rising prevalence of PD and the high healthcare costs further complicate diagnosis, monitoring and management. Consequently, there is a growing effort to find alternative biomarkers that can be tested frequently and remotely to accurately assess PD status [10].

A promising solution is the use of a patient’s voice and speech patterns as biomarkers for disease progression. Speech can be acquired easily and conveniently using smartphones or home devices, enabling remote assessments, anytime and anywhere. This digital acquisition improves accessibility and enables digital analysis in a standard, objective manner. Patient surveys and clinical studies have documented the adverse effects of PD on speech, linking them to dysfunction in the vocal tract muscles. Common characteristics of Parkinsonian speech include a hoarse and breathy voice, monotone delivery, unchanging volume, lack of emotive expression and alterations in intonation and rhythm [11,12,13]. Speech production integrates various physiological subsystems, suggesting that changes may occur early in the disease, even before the onset of motor symptoms [14]. These attributes make speech a good candidate for monitoring PD progression.

Automated speech analysis for PD diagnosis and monitoring is supported by research on speech and voice patterns in conditions such as fatigue and emotional and mental states and in diseases such as laryngeal pathologies and COVID-19 [15,16,17,18,19,20,21]. Leveraging these insights, recent studies employed similar features extracted from PD patients’ recorded speech and utilised machine learning classifiers to estimate disease severity as reflected in the Unified Parkinson’s Disease Rating Scale (UPDRS) score or a Hoehn and Yahr (H&Y) disease stage rating. These features are well-documented voice quality metrics, such as jitter, shimmer, spectral and cepstral features [22,23,24]. Machine learning models, such as support vector machines, regression algorithms and random forests, have shown high accuracy in the diagnosis of PD [25,26]. However, their applicability to clinical settings requires a detailed description of how vocal features change as PD progresses. The existing literature either compares vocal features of Parkinson’s disease patients to healthy controls to identify differences or assesses long-term deterioration by comparing features at different stages of the disease to identify trends.

This paper provides a comprehensive review of vocal features used for longitudinal voice-based assessments of PD severity. It aims to provide an in-depth analysis of how these features change with PD progression. By synthesising existing research, this review seeks to provide valuable insights for biomedical researchers to enhance the accuracy and explainability of automated PD monitoring systems.

This paper is organised as follows: Section 2 provides a summary of related work, providing context for this paper and highlighting the gaps; Section 3 outlines the methods of this review; the results are presented in Section 4; and a discussion of the results and suggestions for future work are presented in Section 5.

## 2. Related Work

Increased interest in using voice as a measure of PD severity provided a growing number of studies and reviews on vocal features. Researchers have examined the effectiveness of various vocal features, feature extraction techniques and pattern recognition methods. The following summary provides an overview of these studies.

Moro-Velazquez et al. [27] reviewed studies investigating the relationship between articulatory and phonatory features of speech and UPDRS scores. They concluded that both feature sets effectively estimate PD severity. Mel-frequency cepstral coefficients (MFCCs), perceptual linear prediction (PLP) coefficients and vowel articulation indicators were used for machine learning algorithms to predict UPDRS scores. Articulatory features (e.g., voice onset time, voice breaks) yielded higher accuracy (80–95%) than phonatory features (e.g., jitter, shimmer), which achieved 75–90% accuracy. The review also noted the importance of methodological control for demographic factors, medication effects and the use of appropriate cross-validation strategies.

Ngo et al. [28] conducted a systematic review of speech and voice analysis for detecting or monitoring PD. The review aimed to determine which speech tasks and acoustic features best reflected disease severity. They found that sustained vowel phonation features were more effective for distinguishing PD from healthy controls, while speaking or reading tasks were better suited for assessing severity or monitoring treatment. Common features included MFCCs, jitter, shimmer, harmonics-to-noise ratio (HNR), fundamental frequency, speech rate and articulatory acoustic vowel space. Support vector machines, random forests, neural networks and deep learning models provided accurate severity estimates, often exceeding 90%. However, results may vary across languages, and missing data points pose challenges for longitudinal studies.

Amato et al. [29] reviewed the application of state-of-the-art analysis methodologies to Parkinsonian speech. The authors concurred with Ngo et al. [28] on the most used acoustic features. MFCCs were found to be particularly effective in the diagnosis of PD and in the estimation of UPDRS scores. The authors emphasised that an inclusion of non-speech factors, such as gender, age, language and time since diagnosis, in the feature sets can enhance model performances.

Reviews by Jones [30] and Moro-Velazquez and Dehak [31] focused on the prosodic aspects of speech. Jones [30] identified the perceptual features of PD as monopitch, monoloudness, reduced intensity and abnormal speaking rate. Acoustic studies support these findings, indicating decreased variability in fundamental frequency and intensity. Moro-Velazquez and Dehak [31] found that PD patients struggle to modify their speech rate and exhibit unorthodox breath pauses while speaking. They also demonstrated that prosodic features alone can support PD diagnosis and monitoring, but combining them with phonatory and articulatory features improves results.

The abovementioned reviews summarise the current research on Parkinsonian voice analysis. However, a significant gap remains in understanding how individual and groups of vocal features change due to PD. Features are identified, but insights into their longitudinal alterations are lacking. This absence of comprehensive acoustic models is documented [27,29] and hinders the development of precise tools for the diagnosis and monitoring of PD. Therefore, a thorough examination of studies analysing Parkinsonian voices is needed to identify those acoustic features and their temporal changes. This study addresses this gap by asking the following: What are the documented changes in vocal features that are associated with PD diagnosis and long-term monitoring?

## 3. Methods

This review used the Preferred Reporting Items for Systematic Reviews and Meta-Analyses (PRISMA) framework to ensure rigour, reliability and reproducibility. The search was conducted in four widely used electronic databases: Scopus, PubMed, Web of Science and IEEE Xplore. The search was limited to English-language articles and conference proceedings published from inception to 31 November 2024.

**Figure 1:**
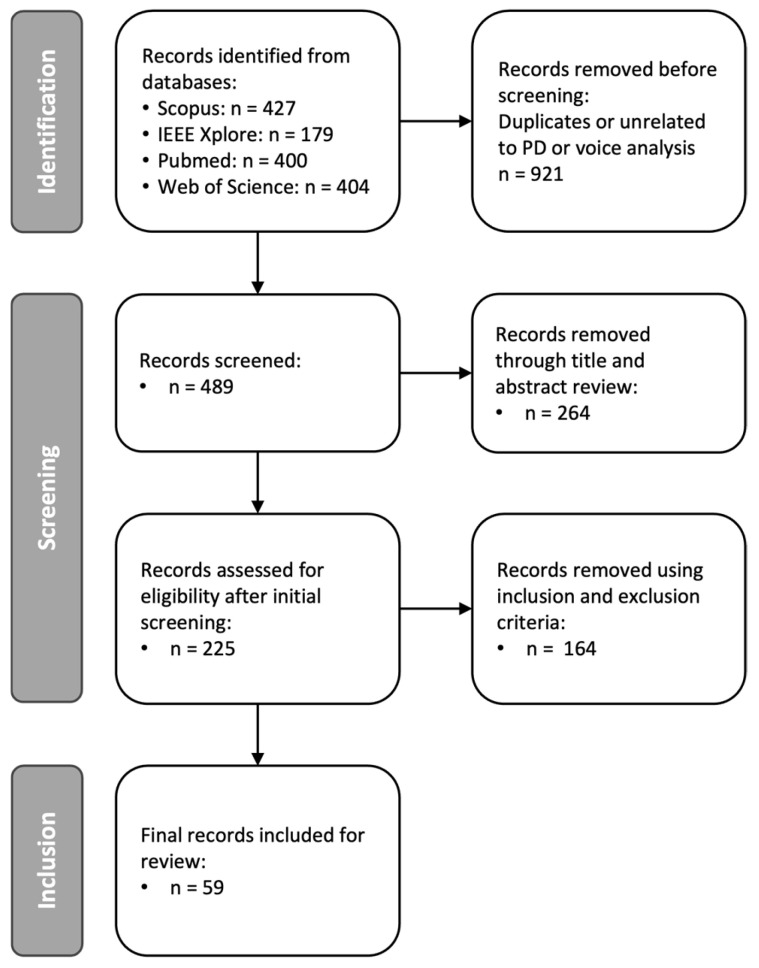
PRISMA review structure implemented for this review.

Relevant works were identified using the following keyword string: [(Parkinson’s disease) AND (monitoring OR estimation OR progression) AND (voice OR speech OR vocal) AND (features OR analysis) AND changes]. To ensure comprehensive capture, synonyms, such as “variations” and “characteristics”, were included. This search string targeted studies on Parkinson’s disease that focus on monitoring, severity estimation or long-term progression of the condition, specifically in relation to voice, speech or vocal aspects. The inclusion of “features”, “analysis” or “characteristics” ensured the capture of studies examining specific attributes of speech or voice changes due to Parkinson’s disease. The search was performed in the title, abstract and keyword fields to maximise the results and recall. The complete search and screening process is illustrated in Figure 1 below.

### 3.1. Study Selection

After completing the initial article search, the titles and abstracts of all retrieved records were screened by the authors manually. No automation tools were used. Full-text articles were assessed for eligibility based on the predefined inclusion criteria listed below:

- English journal articles and conference proceedings analysing voice and speech changes due to Parkinson’s disease.
- Studies describing and assessing measurable changes to the acoustic, phonatory and prosodic characteristics of speech due to PD (as compared to healthy controls).
- Longitudinal studies assessing changes to the acoustic, phonatory and prosodic characteristics of speech due to PD over time.

For the purposes of this review, the following article topics were excluded:

- Studies on PD diagnosis or longitudinal monitoring using other symptoms, such as gait disorders and REM sleep disturbances.
- Studies analysing the effects of medications, therapies or surgeries.
- Review articles on techniques used for PD voice assessment.
- Studies measuring PD progression relative to other rating scales, e.g., the PD composite scale.
- Studies focusing on identifying and analysing cognitive and neurological changes brought on by PD.
- Studies relying solely on self-assessment or perceptual changes to speech and voice.

### 3.2. Data Extraction

The following information was extracted from the selected publications: the primary study aim, type of study (diagnosis or monitoring), data used, the features extracted from the dataset recordings, analysis methods used, noted variations in vocal features and outcomes and results reported. This information was then summarised to identify trends and notable findings, as presented in Section 4 and Section 5.

### 3.3. Search Limitations

Every effort was made to be thorough and inclusive in the search for studies on this niche topic. However, the following limitations were identified:

- Exclusion of other rating scales: While UPDRS and H&Y were chosen for their prevalence in both clinical and research applications, excluding other scales may have limited the findings and the analysis results.
- Language limitation: Including only English-language articles may have resulted in the omission of some findings.
- Exclusion of therapeutic and perceptual assessments: Excluding studies explicitly investigating therapies, perceptual and self-assessments and neurological assessments may have excluded certain results and findings.

## 4. Results

Speech and voice analysis uses a diverse array of features, each offering insights into different pathological changes and having different potential applications. These applications include the PD diagnosis, severity monitoring by estimating a rating scale score and treatment efficacy. In the context of speech production and acoustics, vocal features are commonly grouped into three categories: phonation, articulation and prosody [32,33]. This framework is maintained in our discussion. Table 1 summarises the predominant vocal features in PD monitoring studies and their changes. A detailed examination of these findings follows in subsequent sections.

### 4.1. Phonatory Features

Phonatory features of speech provide information on the functioning of the physiological structures used for breathing and phonation. These include the diaphragm, muscles of the larynx and vocal folds. They are extracted from sustained vowel sounds and can provide insight into pathological changes in the larynx.

#### 4.1.1. Jitter

Jitter is an acoustic feature that measures the variations in the period of consecutive glottal pulses. It may be calculated and represented in several different ways, including absolute jitter, relative percentage jitter and average values calculated using three or more adjacent periods. Due to its ease of evaluation, jitter is one of the most common ones assessed in voice studies and one of the first to be considered for pathological analysis. It has been featured in many classification studies in which features are compared between healthy and diseased voices, and findings from these suggest that the jitter value from a pathological voice would be higher than that from a healthy voice [34,35,49]. However, the exact increase is not quantified. There is also a lack of extended longitudinal studies, which evaluate the changes in jitter values over longer periods of time; thus, further deterioration cannot be quantified.

#### 4.1.2. Shimmer

Shimmer, often studied and reported alongside jitter, is another measure derived from the glottal pulses. It quantifies the difference in amplitudes between consecutive pulses. As with jitter, there are several different ways to calculate and represent it. It has also featured heavily in classification studies, with findings suggesting higher values in pathological voices than in healthy voices [35,48,52]. However, the exact increase has not been quantified, and no long-term longitudinal studies exist to provide details on how shimmer may be affected over the total course of the disease.

#### 4.1.3. Harmonics-to-Noise Ratio

The harmonics-to-noise ratio (HNR) quantifies the amount of noise present in a signal. This may be due to incomplete closure of the vocal cords, which allows for excess air to move through [35]. Since the HNR is a ratio of signal to noise, higher values indicate better signal quality with lower noise content, while lower values mean the opposite. The studies reviewed found that HNR values were lower in PD-affected voices than in healthy voices [49], although longitudinal studies and long-term results are not available. The inverse of the HNR, namely the noise-to-harmonics ratio, is also sometimes reported, showing correspondingly opposite results [34,48,53].

#### 4.1.4. Glottal-to-Noise Excitation Ratio

This feature assesses the relative contributions of vocal fold oscillations and noise to a total voice signal. It is often used in machine learning classifiers to distinguish between PD patients and healthy controls, although the details of the changes in this feature value are not specified. It has not been featured in any longitudinal studies to date [55,56,57].

#### 4.1.5. Correlation Dimension (D2)

Originating in chaotic signal analysis theory, this feature describes the complexity and uncertainty present in vocal sounds. Some studies have identified that an increased D2 value is related to an increase in signal irregularity, and therefore, these values would be higher in pathological voices than in healthy ones [58]. However, it has been noted that D2 is susceptible to spurious noise, such as from recording devices, and, therefore, may not be completely reliable for the task of voice analysis [61].

#### 4.1.6. Pitch Period Entropy

In signal analysis, entropy quantifies signal uncertainty. In speech analysis, these features quantify their inherent complexity. Specifically, pitch period entropy assesses the variability in pitch periods. It has been used in many classification studies, suggesting it is well suited to capturing the changing dynamics of vocal signals [55]. Evidence from two studies shows that the pitch period entropy value in pathological voices may be higher than that of healthy voices [60,61]. However, the long-term changes with worsening PD are unknown.

#### 4.1.7. Recurrent Period Density Entropy

This feature measures signal complexity and uncertainty. It measures how often specified patterns appear in a signal, making it useful for quantifying dysphonia in pathological voices [89,90]. It is often selected for use in diagnostic classifiers, but the values of this feature for healthy and pathological voices have not been quantified. A disadvantage of this feature is that it is complex to interpret and can be difficult to relate back to the original voice signal.

### 4.2. Articulatory Features

These features provide insights into the movements and positioning of the articulatory organs, namely the tongue, lips and jaws, during speech. They offer an understanding of the physical aspects of speech sound production and clarity of speech.

#### 4.2.1. Mel-Frequency Cepstral Coefficients

Cepstral analysis has long been a staple of speech analysis. It allows for the impulse response of the speech system to be isolated from the input and, in so doing, provides a spectral representation of the vocal tract. Mel-frequency cepstral coefficients are most often used to quantify this. They perform well in diagnostic classifiers and are frequently chosen features for this task [62,63,91,92]. However, quantitative comparisons between healthy and PD voices as well as longitudinal analyses are lacking.

#### 4.2.2. Cepstral Peak Prominence

Cepstral peak prominence (CPP) is a feature used for evaluating overall voice quality. It is determined by measuring the height of the cepstrum peak in relation to the baseline levels. It has been widely used in the identification and analysis of speech dysphonia, and it has been investigated specifically for early detection of PD [70] and distinguishing between different PD subtypes [71]. CPP values have been found to be lower in pathological voices than in healthy voices [93], although to date, no long-term data for these values are available. It has also been noted that this feature may be susceptible to background noise present in recordings.

#### 4.2.3. Bark Band Energy Features

Analysing the energy within specific frequency bands of speech signals provides a more detailed understanding of their characteristics. This analysis also aids in understanding how these sounds are perceived by listeners. For PD analysis, Bark band energies are often used. These measure the energy contained in each of 25 frequency bands. These bands are divided according to the Bark scale, which sees the frequency range of audible sound divided into perceptually relevant groups, with smaller bands at lower frequencies and larger bands at higher frequencies. The total amount of energy contained in each band is then calculated. Although they have been used in classifiers to distinguish between PD and healthy voices [72,73], no work has been conducted to compare the energy in each band quantitatively and thus identify where the differences lie and what they are.

#### 4.2.4. Vowel Space Area

The vowel space represents the regions within the mouth where vowel sounds are articulated. In healthy voices, this space is distributed across the entire mouth and palate, allowing for a wide range of vowel sounds to be produced. In PD patients, this vowel space is often diminished, resulting in a more centralised production of vowel sounds. This reduction may be attributed to reduced control of the articulators, such as the tongue, which limits the ability to produce a wide range of vowel sounds.

#### 4.2.5. Vowel Articulation Index

The vowel articulation index is a measure of vowel centralisation and is often studied together with the vowel space area. It is calculated by taking a ratio of the formant frequencies of the vowels /a/ and /i/ to the formant frequencies of the other vowels. It is considered to be robust to inter-speaker variability, thus providing a good general measure for vowel articulation. The values of this feature have been shown to be lower in pathological voices than in healthy voices and to further decrease over time [74,77].

#### 4.2.6. Perceptual Linear Prediction Coefficients

Perceptual linear prediction coefficients provide a representation of the vocal tract in the cepstral domain. This makes them a good choice for tracking changes in articulation [63]. These measures have been used in studies seeking to automatically detect PD, but the differences between them and those calculated for healthy voices are not clarified.

### 4.3. Prosodic Features

These features describe the rhythmic, melodic and expressive aspects of speech. They convey meaning, intention and emotion through speech and extend across whole spoken sentences or passages.

#### 4.3.1. Maximum Phonation Time

This feature indicates how long a person can sustain a phonation sound, such as a vowel. In PD voices, this is reported to be shorter than in healthy voices [83], possibly due to impaired breathing regulation. Exactly how much shorter is uncertain and variable between individual patients and different sounds.

#### 4.3.2. Vocal Pitch Features

Features related to pitch, the voice’s fundamental frequency, quantify the variability in this frequency over a time segment as a measure of the patient’s ability to sustain this single frequency. These features are associated with vocal muscle control, which worsens with progressing PD. They are calculated from the pitch periods observed during sustained vowel phonations. As vocal control in PD patients declines over time and vocal fold oscillations become more erratic, these may become less regular, leading to a change in the fundamental frequency of the voice and increased variability or standard deviation [35,47,48].

Pitch variation may also be assessed over a longer spoken sentence. In this case, the changes to speech prosody are noted, and a reduced variability is observed [94,95,96,97]. This can result in the “monopitch” or “monotone” voice that is reported by both patients and observers of PD speech. It should be noted that these changes are more consistently reported in males than in females.

#### 4.3.3. Speaking Rate

This feature represents the number of sounds a person can produce in a specified time. It is closely associated with the number and length of pauses contained in the spoken recording. It has been shown that the speaking rate is lower in PD patients than in healthy controls, potentially due to patients making a deliberate effort to control their speech [82]. However, to date, no correlation between this feature and PD severity has been identified, and further longitudinal assessments are required to evaluate long-term changes.

#### 4.3.4. Pause Number and Length

The number and length of pauses in a recorded spoken sentence are used to assess the fluency of a patient’s speech. This also affects the rhythm and interpretability of speech. Increased pause times between words or between syllables of words may indicate both decreasing motor control of the speech organs and cognitive decline. This feature is expressed either as a number of pauses that are counted in running speech or a ratio of the total pause length to the total length of recorded speech. In Parkinsonian speech, these feature values tend to be higher than those in healthy speech.

#### 4.3.5. Detrended Fluctuation Analysis

Detrended fluctuation analysis is a statistical parameter used to assess the self-similarity in long speech signals. This is performed by examining the variance in fluctuations over different lengths of a recorded speech segment and determining whether the observed fluctuations are correlated or random. In doing so, regular speaking rhythms can be determined, and unnatural or altered patterns can be identified. This feature may be used to describe the prosodic alterations observed in pathological speech and has been used in classifiers for that purpose. No quantitative data exist on establishing baseline values or distinguishing how pathological values differ from those of healthy ones.

### 4.4. Correlation Between Feature Changes and Progression Rating Scales

Several studies have examined the relationship between vocal feature values and severity scale scores, particularly regarding UPDRS and H&Y scales. Many different aspects of speech have been assessed for this purpose, including vowel articulation [74], syllable repetition stability [87], vocal acoustics during reading tasks and sustained vowels [46,47,48,53], prosodic features [84], speech rate and pause time [85,86] and frequency features.

Despite observing a decline in speech measures over time, the authors found no significant correlations with UPDRS or Hoehn and Yahr scores using statistical tests, such as Pearson correlation and Spearman rank calculations. One study did find a correlation between Hoehn and Yahr scores and perceptual acoustic evaluation but not with UPDRS [51].

The authors propose several possible reasons for the lack of correlation:

- The UPDRS only includes one question related to speech and may not capture the subtle changes seen in vocal features.
- The underlying pathophysiology of speech deterioration may differ from the motor symptoms assessed by the rating scales [74].
- Vocal changes may not be affected by dopaminergic medications, which have the effect of stabilising the UPDRS scores [87].

### 4.5. Feature Impact on Classifier Model Performance

**Figure 2:**
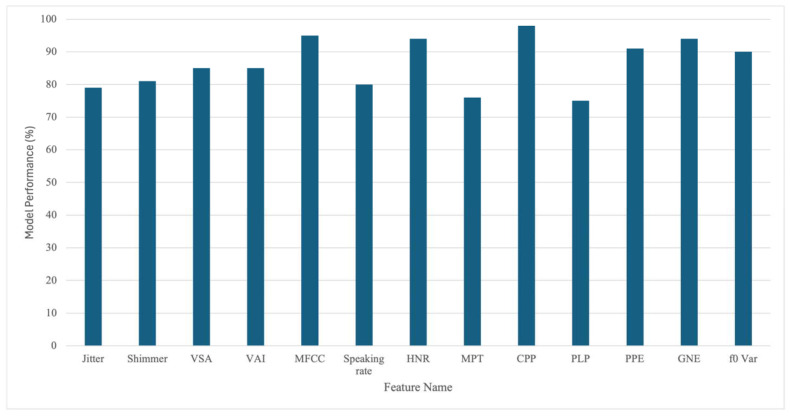
The accuracy of a support vector machine model in Parkinson’s disease prediction using different vocal features, based on results from [28,51,55,61].

To evaluate the effectiveness of the vocal features described above, their impact on models for the diagnosis and monitoring of PD has been examined [28,51,55,61]. Figure 2 summarises the results of these studies and portrays the accuracy of support vector machine (SVM) classifiers in discriminating PD patients from healthy controls using different feature inputs. SVMs are considered here because they are the most frequently used classifiers for this task. The figure demonstrates that diagnostic accuracies above 75% are achieved in all cases, suggesting that vocal features can contribute to the early identification of PD.

It should be noted that the features are not used individually but rather combined into a feature vector, which serves as the input to the machine learning model. While the contribution of individual features is not the focus of this review, understanding their impact on the model’s final output can inform the decisions on which features to include. This feature selection further improves the explainability of the model.

### 4.6. Statistical Approaches in Vocal Feature Studies

The studies reviewed demonstrate several strengths. They consistently apply statistical analysis to compare feature values across different disease stages with healthy controls, highlighting significant differences. The high-performance metrics achieved when using these features in different classification algorithms underscore their utility and merit. Moreover, these works provide objective confirmation of subjectively observed changes, offering a solid foundation for developing a comprehensive acoustic model of vocal deterioration in PD.

However, a notable weakness in this body of literature is the reliance on snapshot measurements, where each patient is recorded at a single point in time. Feature comparison is thus performed on cohorts of patients for each disease stage. This limitation restricts the ability to conduct long-term tracking of individual patients, which is essential for verifying trends in feature values over time. Addressing this weakness would enhance the validity and predictive power of the findings and would contribute to the development of more robust models of disease progression.

The results outlined above highlight the progress made and the ongoing challenges in long-term PD voice analysis, which are discussed in the following section.

## 5. Discussion

This review identified key vocal features used in diagnosing and monitoring Parkinson’s disease and highlighted their changes as the disease progresses. The features examined included both traditional voice quality measures and non-linear characteristics. Classical measures like jitter and shimmer were consistently chosen due to their ease of calculation and explanation. Non-linear signal analysis enables the detection of subtle vocal changes, accommodating the inherent complexity of voice signals. The changes reported are also in line with those described in perceptual evaluations and in the speech section of the UPDRS assessment. Increased noise and signal irregularity relates to the hoarseness or breathy quality of Parkinsonian speech. Imprecise articulation and reduced variation in speaking rates and pitch relate to the monotonic quality, lack of emotion and reduced interpretability of Parkinsonian speech. This underscores the connection between subjective assessments and objective metrics. However, the physiological interpretation of complex non-linear features remains challenging.

Of the reviewed studies, 17 examined vocal changes for the purpose of diagnosing PD, whereas 10 explored monitoring or estimating disease severity. This focus on early diagnosis reflects its clinical importance, enabling earlier treatment, mitigating disease progression and improving patient outcomes. From a research perspective, early diagnostic studies are often more feasible, as vocal features tend to display greater discriminatory capabilities at the outset of the disease compared to the more subtle and diverse changes that emerge as the disease progresses. However, the significance of long-term monitoring, particularly in assessing treatment efficacy, should not be overlooked, especially as novel treatments are introduced. Positive results from studies estimating UPDRS scores or H&Y stages using voice recordings suggest a correlation between these measures, potentially leading to a comprehensive acoustic model of vocal deterioration. Further research is necessary to determine the specific nature of this correlation and to strengthen the utility of voice-based assessments in ongoing disease management.

It should be noted that most studies describe the observed changes in vocal features qualitatively (e.g., “increased” or “decreased”, “statistically significant” or “not statistically significant”). The lack of quantification limits their practical use. Further investigation could determine baseline values for both the disease in general and specific patients, providing more specific objective information regarding the changes and their link to disease progression.

This analysis could be extended to PD subtypes, which are categorised on clinical features. An initial model of vocal deterioration may be used to differentiate Parkinson’s disease from healthy controls. As the model is further refined, it could offer a more detailed examination of each subtype, providing a more nuanced understanding of their distinctive features. Additionally, the inclusion of speech assessments may assist in differential diagnoses between PD and other conditions that present with similar symptoms. These include progressive supranuclear palsy and dementia with Lewy bodies. Studies have shown that speech in dementia with Lewy bodies shows reduced emotional expression [98], while in progressive supranuclear palsy, speakers show reduced articulatory velocity and precision [99]. This suggests that, with further refinement, these tools could be beneficial in the differential diagnosis of these conditions.

Across the reviewed articles, combinations of vocal features, rather than single features in isolation, yielded the most effective diagnostic and monitoring results. This multifaceted approach allows for a comprehensive consideration of vocal changes. However, a notable gap remains in understanding how variations in one vocal feature may influence others. Exploring these interrelationships would enhance the interpretability of vocal features, especially more complex ones. Including metadata, like patient age, gender, time since diagnosis and medications, may allow for more refined models, especially given the current focus on developing patient-centred treatment plans. This could also facilitate the study of longitudinal changes in vocal features, which is notably lacking in many features.

This review revealed a scarcity of long-term analyses, reflecting a broader issue: a lack of accessible data. Most Parkinson’s disease patients are elderly (over 65 years) and may struggle to attend regular clinic visits due to mobility challenges or health issues. Advanced disease stages may also deter participation in studies. Consequently, the studies reviewed often relied on datasets of limited size and availability. Such datasets and a lack of comparative studies restrict the generalisability of the findings. This underscores the need for large-scale longitudinal studies regularly collecting data from the same patients over extended periods. This limitation is exacerbated by the lack of an established experimental protocol. A wide range of feature extraction methods and discrimination models were tested for PD diagnosis and severity estimation. The lack of consistency between them could make it difficult to reach consensus among researchers. Addressing these limitations is crucial to exploiting the promise of machine learning and deep learning models for healthcare applications. Large, consistent datasets for training and validation along with a well-defined experimental and reporting framework would ensure accurate, comparable and explainable results.

Despite these challenges, the substantial research on vocal changes in PD supports the use of voice as a non-invasive and effective biomarker for Parkinson’s disease. The progress in digital speech technologies offers the opportunity for a more objective assessment of voice and speech, enabling precise monitoring over time. The future of vocal evaluation in Parkinson’s disease (PD) includes the integration of these assessments with other PD scales, such as motor and cognitive evaluations, to provide a more comprehensive and patient-centred understanding of disease progression. This holistic approach allows for the development of personalised treatment plans, including pharmaceutical and surgical treatments, as well as speech therapy, which is commonly prescribed to address vocal challenges in PD. This therapy can be efficiently and quantitatively evaluated using digital tools. Moreover, its effects could be assessed not only in the context of improving the patients’ communication abilities but also in potentially slowing cognitive decline and alleviating other disease symptoms. This work fills a critical gap in the existing literature, and integrating these findings into severity prediction models could enhance both their performance and interpretability.

While every effort has been made to ensure a comprehensive review, several limitations exist. For instance, this work examined a limited number of rating scales. The decision to focus on the UPDRS and H&Y scales was based on their widespread use in both research and clinical practice. However, this choice resulted in the exclusion of other rating scales. Including these could provide additional insights into how vocal features change when disease progression is measured using alternative metrics. The decision to exclude perceptual or self-assessments was due to their subjective nature; however, comparing these with objective evaluations of vocal characteristics could yield valuable insights into the psychological effects of the speech changes. Similarly, neurological assessments were not included but could provide additional context for the role of vocal changes in PD progression.

This review only considered changes to voice and speech as PD worsens over time and did not consider the effects of medications or other therapeutic interventions. Thus, potential improvements were not discussed. This represents an additional dimension to vocal changes needing inclusion in any complete model of the effects of PD.

Despite these limitations, this review provides a foundational overview of current research on vocal features as PD biomarkers. Future studies should aim to address them by incorporating additional rating scales and evaluating the effects of medications and therapies.

## 6. Conclusions

Vocal features extracted from speech recordings have emerged as promising, non-invasive biomarkers for PD. This review details how vocal features used in PD monitoring change as the disease progresses. Despite several existing gaps, particularly concerning long-term vocal changes, current insights provide a foundation to predict vocal deterioration in PD patients. These advancements promise more accurate and explainable monitoring methodologies, improving diagnosis and patient care.

Future research should prioritise longitudinal studies capturing long-term vocal changes. Such investigations would not only refine our understanding of how PD affects speech but also provide critical data needed to develop predictive models capable of tracking disease progression accurately. Furthermore, integrating voice-based biomarkers into clinical practice could offer practical benefits, such as early detection, improved treatment planning and enhanced patient monitoring, without the need for invasive procedures.

However, ongoing challenges remain significant. Variability in study methodologies and data quality can complicate efforts to standardise voice-based assessments across different populations or settings. Ensuring accessibility and usability requires collaboration between researchers, clinicians and technology developers to create user-friendly tools for diverse healthcare systems.

Overcoming these challenges through continued research will be crucial to realising the potential of voice-based biomarkers in managing Parkinson’s disease effectively. By fostering interdisciplinary collaboration and investing in further studies on long-term changes in vocal features associated with PD progression, we can move closer to developing robust diagnostic tools that improve patient outcomes while advancing our understanding of this complex neurological disorder.

## Figures and Tables

**Table 1:** Vocal feature descriptions, variations and applications.

Feature Name	Description	Physiological Interpretation	Application	Changes Reported	References
Jitter	Variation in period of glottal pulses	Irregular vocal fold oscillations	Diagnosis; Monitoring	Higher in PD than healthy	[14,34,35,36,37,38,39,40,41,42,43,44,45,46,47,48,49,50,51]
Shimmer	Variation in amplitudes of glottal pulses	Irregular vocal fold oscillations	Diagnosis; Monitoring	Higher in PD than healthy	[14,34,35,36,37,38,39,40,41,42,43,44,45,46,47,48,49,50,51,52,53]
Harmonics-to-Noise Ratio	Ratio between the energy of harmonic content and the energy of the noise content of a signal	Incomplete closure of the vocal folds	Diagnosis; Monitoring	Lower in PD than in healthy	[14,34,35,36,37,39,40,42,43,45,46,47,48,50,51,52,53,54]
Glottal-to-Noise Excitation Ratio	Measure of the contribution of vocal fold oscillations to the voice signal compared to the contribution of noise	Irregular vocal fold oscillations or incomplete closure of the vocal folds	Diagnosis	None recorded	[55,56,57]
Correlation Dimension	Instability of the vocal signal	Irregular vocal fold oscillations	Diagnosis	Higher in PD than in healthy	[58,59]
Pitch Period Entropy	Measure of the variation between pitch periods	Variation in the frequency of vibration of the vocal folds	Diagnosis; Monitoring	Higher in PD than in healthy	[14,36,37,40,42,43,55,60,61]
Recurrent Period Density Entropy	Measure of the complexity of the vocal signal	Irregular vocal fold oscillations	Diagnosis; Monitoring	None recorded	[14,36,37,40,42,43,55,60,61]
Mel-frequency Cepstral Coefficients	Power density spectrum of speech, presented on a perceptually relevant frequency scale	Spectral representation of the vocal tract	Diagnosis; Monitoring	None recorded	[38,45,62,63,64,65,66,67,68,69]
Cepstral Peak Prominence	Height of the cepstral peak in the total cepstrum of a voice signal	Indicator of voice quality	Diagnosis	Lower in PD than in healthy	[54,59,70,71]
Bark Band Energy Features	Measure of the energy contained in each of 25 perceptually relevant frequency bands	Differentiation between voiced and unvoiced segments of speech	Diagnosis	None recorded	[72,73]
Vowel Space Area	A measure of how distinctly different vowel sounds can be produced	Imprecise movements of the articulator organs	Diagnosis; Monitoring	Lower in PD than in healthy	[74,75,76,77,78,79]
Vowel Articulation Index	A measure of where in the mouth vowel sounds are produced	Imprecise movements of the articulator organs	Diagnosis	Lower in PD than in healthy	[74,75,76,77]
Perceptual Linear Prediction Coefficients	Spectral envelope of a speech signal with frequency axis adjusted to the Bark scale	Perceptual representation of the vocal tract	Diagnosis	None recorded	[65,80,81,82]
Maximum Phonation Time	Time that a vowel phonation can be sustained	Illustrates breathing capacity and control	Diagnosis	Shorter in PD than in healthy	[54,83]
Fundamental Frequency Variability	Vocal pitch and its variability	Frequency of the vibration of the vocal folds	Diagnosis; Monitoring	Increased variability over short vocal segments in PD as compared to healthy	[34,35,38,46,47,48,53,54,65,74,84]
Speaking rate	The number of speech sounds produced in a given time	Identification of altered speech patterns	Diagnosis; Monitoring	Lower in PD than in healthy	[53,84,85,86]
Number and Length of Pauses (Pause ratio)	Number and duration of pauses taken during speaking, both between and within words	Identification of altered speech patterns	Monitoring	Higher in PD than in healthy	[53,54,65,84,85,87,88]
Detrended Fluctuation Analysis	Measure of self-similarity and pattern identification in speech signals	Identification of altered speech patterns	Diagnosis	None recorded	[14,36,37,40,42,43,55,60,61]
